# Supplementary material for: Structural brain imaging correlates of general intelligence in UK Biobank
Source: Intelligence. 2019 Sep-Oct;76:101376. doi: 10.1016/j.intell.2019.101376 (PMC6876667; doi:10.1016/j.intell.2019.101376)
Supplement: Supplementary file 2 — Supplementary material. [file mmc2.docx]

**Supplementary Material**

*Table S1.* Self-reported health variables for exclusion criteria

| **Variable** | **Condition** | **Field ID** | **Code** |
| --- | --- | --- | --- |
| *Health* |  |  |  |
| Illness code |  | 20002 |  |
|  | Dementia or Alzheimer’s disease |  | 1263 |
|  | Parkinson’s disease |  | 1262 |
|  | Chronic degenerative neurological |  | 1258 |
|  | Guillain-Barré syndrome |  | 1256 |
|  | Multiple Sclerosis |  | 1261 |
|  | Other demyelinating disease |  | 1397 |
|  | Stroke or ischaemic stroke |  | 1081 |
|  | Brain cancer |  | 1032 |
|  | Brain haemorrhage |  | 1491 |
|  | Brain/intracranial abscess |  | 1245 |
|  | Cerebral aneurysm |  | 1425 |
|  | Cerebral palsy |  | 1433 |
|  | Encephalitis |  | 1246 |
|  | Epilepsy |  | 1264 |
|  | Head injury |  | 1266 |
|  | Infections of the nervous system |  | 1244 |
|  | Ischaemic stroke |  | 1583 |
|  | Meningeal cancer |  | 1031 |
|  | Meningioma (benign) |  | 1659 |
|  | Meningitis |  | 1247 |
|  | Motor Neuron Disease |  | 1259 |
|  | Neurological injury/trauma |  | 1240 |
|  | Spina bifida |  | 1524 |
|  | Subdural haematoma |  | 1083 |
|  | Subarachnoid haemorrhage |  | 1086 |
|  | Transient ischaemic attack |  | 1082 |
| *Cognitive* |  |  |  |
| Symbol-Digit |  | 23324 |  |
| Matrix Reasoning |  | 6373 |  |
| VNR |  | 20016 |  |
| TMTb |  | 6350 |  |
| *Neuroimaging* |  |  |  |
| TBV |  | 25010 |  |
| GM |  | 25006 |  |
| WM |  | 25008 |  |
| WMH |  | 25781 |  |
| Cortical Volumes |  | 25782:25877 |  |
| Subcortical Volumes |  | 25011:25024 |  |
| WM Tract FA |  | 25488:25514 |  |
| WM Tract MD |  | 25515:25541 |  |

*Note.* VNR: verbal numerical reasoning, TMTb: Trail Making Test Part B, TBV: total brain volume, GM: grey matter volume, WM: white matter volume, WMH: white matter hyperintensity volume, FA: fractional anisotropy, MD: mean diffusivity.

*Table S2.* Principal components analysis of the four cognitive tests.

|  | **PC1** | **PC2** | **PC3** | **MR** | **DSS** | **VNR** | **TMTb** |
| --- | --- | --- | --- | --- | --- | --- | --- |
| **Matrix Reasoning** | 0.73 | 0.27 | 0.62 | ***** | 0.360 | 0.405 | -0.302 |
| **Symbol Digit** | 0.72 | -0.57 | -0.01 | <0.001 | ***** | 0.301 | -0.343 |
| **VNR** | 0.71 | 0.53 | -0.41 | <0.001 | <0.001 | ***** | -0.300 |
| **TMTb** | -0.80 | 0.20 | 0.20 | <0.001 | <0.001 | <0.001 | ***** |
| **ProportionVar** | 0.55 | 0.18 | 0.15 | - | - | - | - |
| **CumulativeVar** | 0.55 | 0.73 | 0.88 | - | - | - | - |

*Note.* Loadings of the cognitive tests on the components (left three columns) and correlations among the cognitive tests (Pearson’s *r*; right four columns) are shown. VNR: verbal numerical reasoning, TMTb: Trail Making Test Part B.

*Table S3.* Structural equation model fit statistics for *g* with global MRI measures.

| **Model** | ***χ* ^2^** | **df** | ***p*** | **CFI** | **TLI** | **RMSEA** | **SRMR** |
| --- | --- | --- | --- | --- | --- | --- | --- |
| TBV | 125.775 | 13 | <0.001 | 0.996 | 0.989 | 0.017 | 0.009 |
| GM | 124.275 | 13 | <0.001 | 0.996 | 0.989 | 0.017 | 0.009 |
| NAWM | 117.821 | 13 | <0.001 | 0.996 | 0.989 | 0.017 | 0.009 |
| WMH | 109.909 | 13 | <0.001 | 0.996 | 0.989 | 0.016 | 0.009 |
| *g*FA | 93.175 | 13 | <0.001 | 0.996 | 0.989 | 0.015 | 0.008 |
| *g*MD | 115.748 | 13 | <0.001 | 0.995 | 0.987 | 0.017 | 0.009 |
| *Simultaneous* | 181.005 | 27 | <0.001 | 0.998 | 0.995 | 0.014 | 0.009 |

*Note.* RMSEA: root mean square error of approximation, CFI: comparative fit index, TLI: Tucker Lewis Index, SRMR: standardised root mean square residual. Models are corrected for age and sex; brain measures also corrected for MRI scanner head positioning co-ordinatees. TBV: total brain volume, GM: grey matter volume, WM: white matter volume, WMH: white matter hyperintensity volume, FA: fractional anisotropy, MD: mean diffusivity.

*Table S4.* Residual correlations among global brain imaging measures from SEM Figure 3b.

|  |  | |  | **Std. Est.** | **SE** | ***p*** | **ci.lower** | **ci.upper** |
| --- | --- | --- | --- | --- | --- | --- | --- | --- |
| GM | ~~ | NAWM | | 0.756 | 0.003 | 0.000 | 0.750 | 0.762 |
| NAWM | ~~ | WMH | | 0.069 | 0.005 | 0.000 | 0.060 | 0.078 |
| gFA | ~~ | gMD | | -0.776 | 0.003 | 0.000 | -0.781 | -0.770 |
| GM | ~~ | gFA | | 0.155 | 0.005 | 0.000 | 0.146 | 0.164 |
| NAWM | ~~ | gFA | | 0.120 | 0.006 | 0.000 | 0.108 | 0.131 |
| NAWM | ~~ | gMD | | 0.035 | 0.005 | 0.000 | 0.025 | 0.044 |
| WMH | ~~ | gFA | | -0.321 | 0.007 | 0.000 | -0.333 | -0.308 |
| WMH | ~~ | gMD | | 0.361 | 0.006 | 0.000 | 0.348 | 0.374 |

*Note.* Standardised estimates (Std. Est.), standard errors (SE) are reported with 95% confidence intervals (ci).

*Table S5.* Structural equation model estimates and fit statistics testing factorial invariance of *g* between males and females.

|  | **Baseline** | | **Strong Invariance** | |  |
| --- | --- | --- | --- | --- | --- |
| ***Model Parameters*** | **Females** | **Males** | **Females** | **Males** | **Model**  **Comparison** |
| *g Loadings* |  |  |  |  |  |
| Matrix Reasoning | 1.000 | 1.000 | 1.000 | 1.000 |  |
| Symbol-Digit | 2.335 | 2.372 | 2.356 | 2.356 |  |
| VNR | 1.126 | 1.149 | 1.139 | 1.139 |  |
| Trail Making B | -0.214 | -0.220 | -0.218 | -0.218 |  |
|  |  |  |  |  |  |
| Matrix Reasoning ~~ VNR | 0.511 | 0.232 | 0.522 | 0.226 |  |
|  |  |  |  |  |  |
| *Intercepts* |  |  |  |  |  |
| Matrix Reasoning | 12.352 | 12.983 | 12.614 | 12.614 |  |
| Symbol Digit | 39.294 | 38.757 | 38.842 | 38.842 |  |
| VNR | 8.147 | 8.554 | 8.273 | 8.273 |  |
| Trail Making B | 5.051 | 5.085 | 5.083 | 5.083 |  |
|  |  |  |  |  |  |
| *Model Fit* |  |  |  |  |  |
| CFI |  | 0.998 |  | 0.998 |  |
| TLI |  | 0.984 |  | 0.995 |  |
| RMSEA |  | 0.030 |  | 0.017 |  |
| SRMR |  | 0.005 |  | 0.008 |  |
| AIC |  | 272370 |  | 272370 | 0 |
| saBIC |  | 272544 |  | 272513 | -31 |
| χ^2^ |  | 28.445 |  | 40.266 | 11.821 |
| (df) |  | 2 |  | 8 | 6 |
| *p* |  | <0.001 |  | <0.001 | 0.066 |

*Note.* Unstandardised estimates are reported. VNR: verbal numerical reasoning, CFI: comparative fit index, TLI: Tucker Lewis Index, RMSEA: root mean square error of approximation, SRMR: standardised root mean square residual, AIC: Akaike Information Criterion, saBIC: sample-adjusted Bayesian Information Criterion. Models are corrected for age.

*Table S6.* Associations between *g* and white matter tract fractional anisotropy.

|  | **Std. Est.** | **SE** | ***p*** | **ci.lower** | **ci.upper** | **FDR *q*** |
| --- | --- | --- | --- | --- | --- | --- |
| lAR | 0.021 | 0.011 | 0.064 | -0.001 | 0.043 | 0.066 |
| rAR | 0.023 | 0.011 | 0.045 | 0.000 | 0.045 | 0.049 |
| lATR | 0.089 | 0.011 | <0.001 | 0.067 | 0.112 | <0.001 |
| rATR | 0.090 | 0.011 | <0.001 | 0.068 | 0.112 | <0.001 |
| lCingG | 0.032 | 0.011 | 0.005 | 0.010 | 0.054 | 0.006 |
| rCingG | 0.036 | 0.011 | 0.001 | 0.014 | 0.058 | 0.002 |
| lCingPH | 0.044 | 0.011 | <0.001 | 0.023 | 0.066 | <0.001 |
| rCingPH | 0.025 | 0.011 | 0.023 | 0.003 | 0.047 | 0.026 |
| lCST | 0.032 | 0.011 | 0.004 | 0.010 | 0.054 | 0.005 |
| rCST | 0.044 | 0.011 | <0.001 | 0.022 | 0.066 | <0.001 |
| FMaj | 0.063 | 0.011 | <0.001 | 0.041 | 0.085 | <0.001 |
| FMin | 0.066 | 0.011 | <0.001 | 0.043 | 0.088 | <0.001 |
| lIFOF | 0.083 | 0.011 | <0.001 | 0.061 | 0.105 | <0.001 |
| rIFOF | 0.065 | 0.011 | <0.001 | 0.043 | 0.087 | <0.001 |
| lILF | 0.084 | 0.011 | <0.001 | 0.062 | 0.106 | <0.001 |
| rILF | 0.076 | 0.011 | <0.001 | 0.054 | 0.098 | <0.001 |
| MCP | 0.012 | 0.011 | 0.288 | -0.010 | 0.034 | 0.288 |
| lML | 0.059 | 0.011 | <0.001 | 0.037 | 0.080 | <0.001 |
| rML | 0.057 | 0.011 | <0.001 | 0.035 | 0.079 | <0.001 |
| lPTR | 0.110 | 0.011 | <0.001 | 0.088 | 0.132 | <0.001 |
| rPTR | 0.097 | 0.011 | <0.001 | 0.075 | 0.119 | <0.001 |
| lSLF | 0.069 | 0.011 | <0.001 | 0.047 | 0.092 | <0.001 |
| rSLF | 0.075 | 0.011 | <0.001 | 0.053 | 0.097 | <0.001 |
| lSTR | 0.038 | 0.011 | 0.001 | 0.017 | 0.060 | 0.001 |
| rSTR | 0.042 | 0.011 | <0.001 | 0.021 | 0.064 | <0.001 |
| lUnc | 0.079 | 0.011 | <0.001 | 0.056 | 0.101 | <0.001 |
| rUnc | 0.074 | 0.011 | <0.001 | 0.052 | 0.096 | <0.001 |

*Note.* Standardised estimates (Std. Est.), standard errors (SE) are reported. Right and left are denoted by l and r. AR: acoustic radiation, ATR: anterior thalamic radiation, Cing: cingulum bundle gyrus (G) and parahippocampal (PH), CST: corticospinal tract, FMaj: forceps major, FMin: forceps minor, IFOF: inferior fronto-occipital fasciculus, ILF: inferior longitudinal fasciculus, MCP: middle cerebellar peduncle, ML: medial lemniscus, PTR: posterior thalamic radiation, SLF: superior longitudinal fasciculus, STR: superior thalamic radiation, Unc: uncinate. All model fits were χ^2^ (13) ≤ 101.831 *p* < 0.001, CFI ≥ 0.995, TLI ≥ 0.987, RMSEA ≤ 0.015, SRMR ≤ 0.009.

*Table S7.* Associations between *g* and white matter tract mean diffusivity.

|  | **Std. Est.** | **SE** | ***p*** | **ci.lower** | **ci.upper** | **FDR *q*** |
| --- | --- | --- | --- | --- | --- | --- |
| lAR | 0.003 | 0.011 | 0.804 | -0.019 | 0.025 | 0.804 |
| rAR | -0.026 | 0.011 | 0.018 | -0.048 | -0.005 | 0.022 |
| lATR | -0.089 | 0.013 | 0.000 | -0.114 | -0.064 | 0.000 |
| rATR | -0.093 | 0.013 | 0.000 | -0.119 | -0.068 | 0.000 |
| lCingG | -0.039 | 0.012 | 0.001 | -0.062 | -0.015 | 0.002 |
| rCingG | -0.052 | 0.012 | 0.000 | -0.076 | -0.028 | 0.000 |
| lCingPH | -0.033 | 0.011 | 0.003 | -0.055 | -0.011 | 0.004 |
| rCingPH | -0.008 | 0.011 | 0.478 | -0.030 | 0.014 | 0.516 |
| lCST | -0.045 | 0.011 | 0.000 | -0.067 | -0.023 | 0.000 |
| rCST | -0.065 | 0.011 | 0.000 | -0.087 | -0.043 | 0.000 |
| FMaj | -0.016 | 0.012 | 0.162 | -0.039 | 0.006 | 0.182 |
| FMin | -0.052 | 0.012 | 0.000 | -0.075 | -0.028 | 0.000 |
| lIFOF | -0.055 | 0.012 | 0.000 | -0.078 | -0.031 | 0.000 |
| rIFOF | -0.044 | 0.012 | 0.000 | -0.067 | -0.021 | 0.000 |
| lILF | -0.046 | 0.012 | 0.000 | -0.070 | -0.022 | 0.000 |
| rILF | -0.038 | 0.012 | 0.002 | -0.061 | -0.014 | 0.002 |
| MCP | -0.055 | 0.011 | 0.000 | -0.077 | -0.032 | 0.000 |
| lML | -0.019 | 0.011 | 0.078 | -0.041 | 0.002 | 0.092 |
| rML | 0.007 | 0.011 | 0.511 | -0.014 | 0.029 | 0.531 |
| lPTR | -0.077 | 0.012 | 0.000 | -0.100 | -0.053 | 0.000 |
| rPTR | -0.093 | 0.012 | 0.000 | -0.117 | -0.069 | 0.000 |
| lSLF | -0.045 | 0.012 | 0.000 | -0.068 | -0.022 | 0.000 |
| rSLF | -0.055 | 0.012 | 0.000 | -0.079 | -0.032 | 0.000 |
| lSTR | -0.092 | 0.013 | 0.000 | -0.117 | -0.067 | 0.000 |
| rSTR | -0.100 | 0.013 | 0.000 | -0.126 | -0.074 | 0.000 |
| lUnc | -0.097 | 0.013 | 0.000 | -0.122 | -0.072 | 0.000 |
| rUnc | -0.083 | 0.012 | 0.000 | -0.106 | -0.059 | 0.000 |

*Note.* Standardised estimates, standard errors (SE) are reported. AR: acoustic radiation, ATR: anterior thalamic radiation, Cing: cingulum bundle gyrus (G) and parahippocampal (PH), CST: corticospinal tract, FMaj: forceps major, FMin: forceps minor, IFOF: inferior fronto-occipital fasciculus, ILF: inferior longitudinal fasciculus, MCP: middle cerebellar peduncle, ML: medial lemniscus, PTR: posterior thalamic radiation, SLF: superior longitudinal fasciculus, STR: superior thalamic radiation, Unc: uncinate. All model fits were χ^2^ (13) ≤ 117.848 *p* < 0.001, CFI ≥ 0.995, TLI ≥ 0.986, RMSEA ≤ 0.017, SRMR ≤ 0.010.

*Table S8.* Associations between *g* and grey matter regional volumes.

|  | **Std. Est.** | **SE** | ***p*** | **ci.lower** | **ci.upper** |
| --- | --- | --- | --- | --- | --- |
| FrontalPoleleft | 0.186 | 0.012 | 0.000 | 0.163 | 0.208 |
| FrontalPoleright | 0.216 | 0.011 | 0.000 | 0.194 | 0.239 |
| InsularCortexleft | 0.194 | 0.011 | 0.000 | 0.172 | 0.215 |
| InsularCortexright | 0.205 | 0.011 | 0.000 | 0.183 | 0.226 |
| SuperiorFrontalGyrusleft | 0.123 | 0.011 | 0.000 | 0.102 | 0.144 |
| SuperiorFrontalGyrusright | 0.121 | 0.011 | 0.000 | 0.099 | 0.142 |
| MiddleFrontalGyrusleft | 0.127 | 0.011 | 0.000 | 0.106 | 0.148 |
| MiddleFrontalGyrusright | 0.101 | 0.011 | 0.000 | 0.080 | 0.123 |
| InferiorFrontalGyrusparstriangularisleft | 0.064 | 0.011 | 0.000 | 0.043 | 0.085 |
| InferiorFrontalGyrusparstriangularisright | 0.069 | 0.011 | 0.000 | 0.048 | 0.089 |
| InferiorFrontalGyrusparsopercularisleft | 0.091 | 0.011 | 0.000 | 0.070 | 0.112 |
| InferiorFrontalGyrusparsopercularisright | 0.062 | 0.011 | 0.000 | 0.041 | 0.083 |
| PrecentralGyrusleft | 0.165 | 0.011 | 0.000 | 0.144 | 0.187 |
| PrecentralGyrusright | 0.161 | 0.011 | 0.000 | 0.139 | 0.183 |
| TemporalPoleleft | 0.151 | 0.011 | 0.000 | 0.128 | 0.173 |
| TemporalPoleright | 0.151 | 0.011 | 0.000 | 0.129 | 0.173 |
| SuperiorTemporalGyrusanteriorleft | 0.139 | 0.011 | 0.000 | 0.118 | 0.160 |
| SuperiorTemporalGyrusanteriorright | 0.141 | 0.011 | 0.000 | 0.120 | 0.162 |
| SuperiorTemporalGyrusposteriorleft | 0.117 | 0.011 | 0.000 | 0.095 | 0.138 |
| SuperiorTemporalGyrusposteriorright | 0.119 | 0.011 | 0.000 | 0.097 | 0.140 |
| MiddleTemporalGyrusanteriorleft | 0.128 | 0.011 | 0.000 | 0.107 | 0.149 |
| MiddleTemporalGyrusanteriorright | 0.116 | 0.011 | 0.000 | 0.095 | 0.138 |
| MiddleTemporalGyrusposteriorleft | 0.102 | 0.011 | 0.000 | 0.080 | 0.123 |
| MiddleTemporalGyrusposteriorright | 0.107 | 0.011 | 0.000 | 0.086 | 0.129 |
| MiddleTemporalGyrustemporooccipitalleft | 0.070 | 0.010 | 0.000 | 0.049 | 0.090 |
| MiddleTemporalGyrustemporooccipitalright | 0.086 | 0.011 | 0.000 | 0.064 | 0.107 |
| InferiorTemporalGyrusanteriorleft | 0.071 | 0.011 | 0.000 | 0.050 | 0.092 |
| InferiorTemporalGyrusanteriorright | 0.082 | 0.011 | 0.000 | 0.061 | 0.104 |
| InferiorTemporalGyrusposteriorleft | 0.077 | 0.011 | 0.000 | 0.055 | 0.098 |
| InferiorTemporalGyrusposteriorright | 0.081 | 0.011 | 0.000 | 0.059 | 0.102 |
| InferiorTemporalGyrustemporooccipitalleft | 0.062 | 0.011 | 0.000 | 0.041 | 0.084 |
| InferiorTemporalGyrustemporooccipitalright | 0.074 | 0.011 | 0.000 | 0.052 | 0.096 |
| PostcentralGyrusleft | 0.116 | 0.011 | 0.000 | 0.095 | 0.137 |
| PostcentralGyrusright | 0.124 | 0.011 | 0.000 | 0.103 | 0.146 |
| SuperiorParietalLobuleleft | 0.066 | 0.011 | 0.000 | 0.045 | 0.087 |
| SuperiorParietalLobuleright | 0.075 | 0.011 | 0.000 | 0.055 | 0.096 |
| SupramarginalGyrusanteriorleft | 0.068 | 0.011 | 0.000 | 0.047 | 0.089 |
| SupramarginalGyrusanteriorright | 0.092 | 0.010 | 0.000 | 0.071 | 0.112 |
| SupramarginalGyrusposteriorleft | 0.083 | 0.011 | 0.000 | 0.062 | 0.104 |
| SupramarginalGyrusposteriorright | 0.098 | 0.011 | 0.000 | 0.078 | 0.119 |
| AngularGyrusleft | 0.079 | 0.010 | 0.000 | 0.058 | 0.099 |
| AngularGyrusright | 0.100 | 0.011 | 0.000 | 0.079 | 0.121 |
| LateralOccipitalCortexsuperiorleft | 0.139 | 0.011 | 0.000 | 0.117 | 0.160 |
| LateralOccipitalCortexsuperiorright | 0.117 | 0.011 | 0.000 | 0.095 | 0.139 |
| LateralOccipitalCortexinferiorleft | 0.118 | 0.011 | 0.000 | 0.097 | 0.139 |
| LateralOccipitalCortexinferiorright | 0.110 | 0.011 | 0.000 | 0.088 | 0.132 |
| IntracalcarineCortexleft | 0.078 | 0.011 | 0.000 | 0.057 | 0.100 |
| IntracalcarineCortexright | 0.093 | 0.011 | 0.000 | 0.072 | 0.114 |
| FrontalMedialCortexleft | 0.107 | 0.011 | 0.000 | 0.086 | 0.128 |
| FrontalMedialCortexright | 0.104 | 0.011 | 0.000 | 0.083 | 0.125 |
| JuxtapositionalLobuleCortex | 0.090 | 0.011 | 0.000 | 0.069 | 0.111 |
| JuxtapositionalLobuleCortex | 0.095 | 0.011 | 0.000 | 0.074 | 0.116 |
| SubcallosalCortexleft | 0.175 | 0.011 | 0.000 | 0.153 | 0.197 |
| SubcallosalCortexright | 0.166 | 0.011 | 0.000 | 0.144 | 0.188 |
| ParacingulateGyrusleft | 0.134 | 0.011 | 0.000 | 0.112 | 0.156 |
| ParacingulateGyrusright | 0.128 | 0.011 | 0.000 | 0.106 | 0.149 |
| CingulateGyrusanteriorleft | 0.048 | 0.011 | 0.000 | 0.027 | 0.069 |
| CingulateGyrusanteriorright | 0.053 | 0.011 | 0.000 | 0.032 | 0.074 |
| CingulateGyrusposteriorleft | 0.145 | 0.012 | 0.000 | 0.122 | 0.168 |
| CingulateGyrusposteriorright | 0.134 | 0.011 | 0.000 | 0.112 | 0.157 |
| PrecuneousCortexleft | 0.135 | 0.011 | 0.000 | 0.113 | 0.156 |
| PrecuneousCortexright | 0.138 | 0.011 | 0.000 | 0.116 | 0.160 |
| CunealCortexleft | 0.065 | 0.011 | 0.000 | 0.044 | 0.086 |
| CunealCortexright | 0.087 | 0.011 | 0.000 | 0.065 | 0.108 |
| FrontalOrbitalCortexleft | 0.174 | 0.011 | 0.000 | 0.151 | 0.196 |
| FrontalOrbitalCortexright | 0.174 | 0.011 | 0.000 | 0.152 | 0.196 |
| ParahippocampalGyrusanteriorleft | 0.146 | 0.011 | 0.000 | 0.124 | 0.168 |
| ParahippocampalGyrusanteriorright | 0.143 | 0.011 | 0.000 | 0.121 | 0.165 |
| ParahippocampalGyrusposteriorleft | 0.104 | 0.011 | 0.000 | 0.082 | 0.125 |
| ParahippocampalGyrusposteriorright | 0.087 | 0.011 | 0.000 | 0.066 | 0.108 |
| LingualGyrusleft | 0.140 | 0.012 | 0.000 | 0.117 | 0.164 |
| LingualGyrusright | 0.156 | 0.012 | 0.000 | 0.133 | 0.179 |
| TemporalFusiformCortexanteriorleft | 0.147 | 0.011 | 0.000 | 0.125 | 0.168 |
| TemporalFusiformCortexanteriorright | 0.146 | 0.011 | 0.000 | 0.124 | 0.168 |
| TemporalFusiformCortexposteriorleft | 0.152 | 0.011 | 0.000 | 0.129 | 0.174 |
| TemporalFusiformCortexposteriorright | 0.147 | 0.011 | 0.000 | 0.125 | 0.169 |
| TemporalOccipitalFusiformCortexleft | 0.088 | 0.011 | 0.000 | 0.067 | 0.109 |
| TemporalOccipitalFusiformCortexright | 0.114 | 0.011 | 0.000 | 0.093 | 0.136 |
| OccipitalFusiformGyrusleft | 0.101 | 0.011 | 0.000 | 0.080 | 0.123 |
| OccipitalFusiformGyrusright | 0.112 | 0.011 | 0.000 | 0.090 | 0.133 |
| FrontalOperculumCortexleft | 0.128 | 0.011 | 0.000 | 0.106 | 0.149 |
| FrontalOperculumCortexright | 0.101 | 0.011 | 0.000 | 0.080 | 0.123 |
| CentralOpercularCortexleft | 0.171 | 0.011 | 0.000 | 0.149 | 0.193 |
| CentralOpercularCortexright | 0.151 | 0.011 | 0.000 | 0.129 | 0.173 |
| ParietalOperculumCortexleft | 0.107 | 0.011 | 0.000 | 0.086 | 0.129 |
| ParietalOperculumCortexright | 0.108 | 0.011 | 0.000 | 0.086 | 0.130 |
| PlanumPolareleft | 0.131 | 0.011 | 0.000 | 0.109 | 0.152 |
| PlanumPolareright | 0.154 | 0.011 | 0.000 | 0.132 | 0.176 |
| HeschlsGyrusincludesH1andH2left | 0.120 | 0.011 | 0.000 | 0.098 | 0.142 |
| HeschlsGyrusincludesH1andH2right | 0.141 | 0.011 | 0.000 | 0.119 | 0.163 |
| PlanumTemporaleleft | 0.118 | 0.011 | 0.000 | 0.096 | 0.140 |
| PlanumTemporaleright | 0.130 | 0.011 | 0.000 | 0.108 | 0.152 |
| SupracalcarineCortexleft | 0.073 | 0.011 | 0.000 | 0.052 | 0.094 |
| SupracalcarineCortexright | 0.081 | 0.011 | 0.000 | 0.060 | 0.102 |
| OccipitalPoleleft | 0.093 | 0.011 | 0.000 | 0.071 | 0.115 |
| OccipitalPoleright | 0.080 | 0.011 | 0.000 | 0.057 | 0.102 |

*Note.* Standardised estimates (Std. Est.), standard errors (SE) are reported. All model fits were χ^2^ (13) ≤ 134.92 *p* < 0.001, CFI ≥ 0.995, TLI ≥ 0.985, RMSEA ≤ 0.018, SRMR ≤ 0.010.

*Table S9.* Associations between *g* and subcortical volumes.

|  | **Std. Est.** | **SE** | ***p*** | **ci.lower** | **ci.upper** |
| --- | --- | --- | --- | --- | --- |
| lAccumbens | 0.113 | 0.011 | <0.001 | 0.091 | 0.135 |
| rAccumbens | 0.105 | 0.011 | <0.001 | 0.083 | 0.128 |
| lAmygdala | 0.075 | 0.011 | <0.001 | 0.054 | 0.096 |
| rAmygdala | 0.062 | 0.011 | <0.001 | 0.041 | 0.083 |
| lCaudate | 0.131 | 0.011 | <0.001 | 0.109 | 0.152 |
| rCaudate | 0.126 | 0.011 | <0.001 | 0.105 | 0.147 |
| lHippocampus | 0.131 | 0.011 | <0.001 | 0.110 | 0.152 |
| rHippocampus | 0.150 | 0.011 | <0.001 | 0.129 | 0.171 |
| lPallidum | 0.141 | 0.011 | <0.001 | 0.119 | 0.162 |
| rPallidum | 0.142 | 0.011 | <0.001 | 0.120 | 0.163 |
| lPutamen | 0.169 | 0.012 | <0.001 | 0.146 | 0.192 |
| rPutamen | 0.165 | 0.012 | <0.001 | 0.142 | 0.188 |
| lThalamus | 0.251 | 0.012 | <0.001 | 0.229 | 0.274 |
| rThalamus | 0.256 | 0.012 | <0.001 | 0.233 | 0.279 |

*Note.* Standardised estimates (Std. Est.), standard errors (SE) are reported with 95% confidence intervals (ci). All model fits were χ^2^ (13) ≤ 137.941 *p* < 0.001, CFI ≥ 0.994, TLI ≥ 0.983, RMSEA ≤ 0.018, SRMR ≤ 0.011.

Table S10. Standardised beta weights for unique contributions of white matter tract fractional anisotropy to variance in *g,* estimated from a MIMIC model in the training set (Manchester).

|  | **Est.** | **SE** | ***p*** | **ci.lower** | **ci.upper** |
| --- | --- | --- | --- | --- | --- |
| SLF | 0.028 | 0.023 | 0.229 | -0.017 | 0.072 |
| AR | -0.060 | 0.018 | 0.001 | -0.095 | -0.025 |
| ATR | 0.074 | 0.022 | 0.001 | 0.031 | 0.117 |
| CingG | -0.005 | 0.015 | 0.753 | -0.033 | 0.024 |
| CST | 0.002 | 0.018 | 0.892 | -0.033 | 0.038 |
| FMaj | 0.019 | 0.015 | 0.218 | -0.011 | 0.049 |
| FMin | -0.022 | 0.022 | 0.305 | -0.065 | 0.020 |
| ILF | -0.001 | 0.034 | 0.984 | -0.067 | 0.066 |
| IFOF | -0.082 | 0.033 | 0.012 | -0.146 | -0.018 |
| PTR | 0.128 | 0.023 | <0.001 | 0.083 | 0.173 |
| STR | -0.032 | 0.020 | 0.116 | -0.071 | 0.008 |
| Unc | 0.067 | 0.018 | <0.001 | 0.033 | 0.102 |
| ML | 0.053 | 0.014 | <0.001 | 0.026 | 0.081 |
| MCP | -0.013 | 0.013 | 0.348 | -0.039 | 0.014 |
| CingPH | -0.002 | 0.013 | 0.877 | -0.028 | 0.024 |

Fits for the initial SEM in Manchester sample were: χ^2^ (62) = 1649.592, *p* < 0.001, CFI = 0.991, TLI = 0.960, RMSEA = 0.034, SRMR = 0.042.

Table S11. Standardised beta weights for unique contributions of white matter tract mean diffusivity to variance in *g,* estimated from a MIMIC model in the training set (Manchester).

|  | **Est.** | **SE** | ***p*** | **ci.lower** | **ci.upper** |
| --- | --- | --- | --- | --- | --- |
| SLF | 0.015 | 0.032 | 0.636 | -0.047 | 0.077 |
| AR | 0.023 | 0.016 | 0.151 | -0.008 | 0.054 |
| ATR | -0.026 | 0.027 | 0.341 | -0.078 | 0.027 |
| CingG | 0.002 | 0.023 | 0.929 | -0.043 | 0.047 |
| CST | -0.015 | 0.017 | 0.404 | -0.049 | 0.020 |
| FMaj | -0.002 | 0.015 | 0.907 | -0.032 | 0.028 |
| FMin | 0.012 | 0.022 | 0.594 | -0.031 | 0.054 |
| ILF | 0.225 | 0.043 | <0.001 | 0.140 | 0.310 |
| IFOF | 0.032 | 0.043 | 0.455 | -0.052 | 0.115 |
| PTR | -0.177 | 0.023 | <0.001 | -0.222 | -0.133 |
| STR | -0.095 | 0.031 | 0.002 | -0.156 | -0.034 |
| Unc | -0.167 | 0.024 | <0.001 | -0.213 | -0.120 |
| ML | 0.020 | 0.013 | 0.136 | -0.006 | 0.046 |
| MCP | -0.030 | 0.013 | 0.024 | -0.056 | -0.004 |
| CingPH | 0.005 | 0.013 | 0.687 | -0.021 | 0.031 |

Fits for the initial SEM in Manchester sample were: χ^2^ (66) = 2015.433, *p* < 0.001, CFI = 0.991, TLI = 0.965, RMSEA = 0.037, SRMR = 0.049.

Table S12. Standardised beta weights for unique contributions of regional cortical volumes to variance in *g,* estimated from a MIMIC model in the training set (Manchester).

|  | **Est** | **SE** | ***p*** | **ci.lower** | **ci.upper** |
| --- | --- | --- | --- | --- | --- |
| FrontalPole | 0.062 | 0.022 | 0.006 | 0.017 | 0.106 |
| InsularCortex | 0.056 | 0.021 | 0.008 | 0.015 | 0.098 |
| SuperiorFrontalGyrus | -0.015 | 0.015 | 0.318 | -0.046 | 0.015 |
| MiddleFrontalGyrus | 0.006 | 0.016 | 0.710 | -0.026 | 0.038 |
| InferiorFrontalGyrusparstriangularis | -0.021 | 0.016 | 0.201 | -0.053 | 0.011 |
| InferiorFrontalGyrusparsopercularis | 0.012 | 0.014 | 0.419 | -0.017 | 0.04 |
| PrecentralGyrus | 0.047 | 0.017 | 0.007 | 0.013 | 0.081 |
| TemporalPole | -0.014 | 0.019 | 0.465 | -0.050 | 0.023 |
| SuperiorTemporalGyrusanterior | 0.064 | 0.017 | <0.001 | 0.030 | 0.098 |
| SuperiorTemporalGyrusposterior | 0.003 | 0.020 | 0.865 | -0.035 | 0.042 |
| MiddleTemporalGyrusanterior | 0.025 | 0.017 | 0.145 | -0.009 | 0.058 |
| MiddleTemporalGyrusposterior | -0.033 | 0.019 | 0.079 | -0.070 | 0.004 |
| MiddleTemporalGyrustemporooccipital | 0.016 | 0.016 | 0.311 | -0.015 | 0.046 |
| InferiorTemporalGyrusanterior | -0.002 | 0.016 | 0.882 | -0.034 | 0.029 |
| InferiorTemporalGyrusposterior | -0.033 | 0.019 | 0.094 | -0.071 | 0.006 |
| InferiorTemporalGyrustemporooccipital | -0.025 | 0.015 | 0.098 | -0.055 | 0.005 |
| PostcentralGyrus | 0.009 | 0.018 | 0.603 | -0.025 | 0.043 |
| SuperiorParietalLobule | -0.014 | 0.013 | 0.289 | -0.040 | 0.012 |
| SupramarginalGyrusanterior | -0.009 | 0.016 | 0.596 | -0.041 | 0.023 |
| SupramarginalGyrusposterior | 0.023 | 0.017 | 0.169 | -0.010 | 0.057 |
| AngularGyrus | 0.019 | 0.015 | 0.215 | -0.011 | 0.049 |
| LateralOccipitalCortexsuperior | 0.003 | 0.016 | 0.837 | -0.029 | 0.036 |
| LateralOccipitalCortexinferior | 0.007 | 0.016 | 0.674 | -0.025 | 0.039 |
| IntracalcarineCortex | 0.038 | 0.017 | 0.029 | 0.004 | 0.072 |
| FrontalMedialCortex | 0.015 | 0.014 | 0.262 | -0.011 | 0.042 |
| JuxtapositionalLobuleCortex | -0.002 | 0.013 | 0.879 | -0.028 | 0.024 |
| SubcallosalCortex | 0.037 | 0.018 | 0.039 | 0.002 | 0.072 |
| ParacingulateGyrus | -0.003 | 0.016 | 0.859 | -0.035 | 0.029 |
| CingulateGyrusanterior | -0.041 | 0.014 | 0.005 | -0.069 | -0.013 |
| CingulateGyrusposterior | -0.004 | 0.017 | 0.835 | -0.037 | 0.030 |
| PrecuneousCortex | -0.020 | 0.018 | 0.275 | -0.056 | 0.016 |
| CunealCortex | -0.032 | 0.017 | 0.062 | -0.065 | 0.002 |
| FrontalOrbitalCortex | 0.029 | 0.019 | 0.129 | -0.008 | 0.065 |
| ParahippocampalGyrusanterior | 0.021 | 0.017 | 0.218 | -0.012 | 0.054 |
| ParahippocampalGyrusposterior | 0.021 | 0.014 | 0.115 | -0.005 | 0.048 |
| LingualGyrus | 0.012 | 0.018 | 0.496 | -0.023 | 0.047 |
| TemporalFusiformCortexanterior | 0.053 | 0.018 | 0.004 | 0.017 | 0.089 |
| TemporalFusiformCortexposterior | 0.040 | 0.02 | 0.045 | 0.001 | 0.079 |
| TemporalOccipitalFusiformCortex | 0.004 | 0.016 | 0.821 | -0.028 | 0.035 |
| OccipitalFusiformGyrus | 0.009 | 0.016 | 0.571 | -0.023 | 0.042 |
| FrontalOperculumCortex | 0.027 | 0.019 | 0.157 | -0.010 | 0.063 |
| CentralOpercularCortex | 0.001 | 0.021 | 0.954 | -0.041 | 0.043 |
| ParietalOperculumCortex | 0.010 | 0.021 | 0.631 | -0.031 | 0.052 |
| PlanumPolare | -0.021 | 0.020 | 0.292 | -0.060 | 0.018 |
| HeschlsGyrusincludesH1andH2 | 0.018 | 0.023 | 0.416 | -0.026 | 0.062 |
| PlanumTemporale | 0.011 | 0.029 | 0.690 | -0.045 | 0.068 |
| SupracalcarineCortex | 0.003 | 0.017 | 0.846 | -0.030 | 0.037 |
| OccipitalPole | -0.027 | 0.018 | 0.124 | -0.061 | 0.007 |

Fits for the initial SEM in Manchester sample were: χ^2^ (164) = 1820.630, *p* < 0.001, CFI = 0.997, TLI = 0.973, RMSEA = 0.021, SRMR = 0.038.

Table S13. Standardised beta weights for unique contributions of subcortical volumes to variance in *g,* estimated from a MIMIC model in the training set (Manchester).

|  | **Est.** | **SE** | ***p*** | **ci.lower** | **ci.upper** |
| --- | --- | --- | --- | --- | --- |
| Accumbens | -0.009 | 0.015 | 0.551 | -0.038 | 0.020 |
| Amygdala | 0.008 | 0.013 | 0.504 | -0.016 | 0.033 |
| Caudate | 0.019 | 0.014 | 0.179 | -0.009 | 0.048 |
| HC | 0.048 | 0.015 | 0.001 | 0.019 | 0.076 |
| Pallidum | 0.024 | 0.015 | 0.125 | -0.007 | 0.054 |
| Putamen | 0.038 | 0.017 | 0.029 | 0.004 | 0.071 |
| Thalamus | 0.194 | 0.019 | <0.001 | 0.157 | 0.230 |

Fits for the initial SEM in Manchester sample were: χ^2^ (31) = 105.332, *p* < 0.001, CFI = 0.999, TLI = 0.996, RMSEA = 0.010, SRMR = 0.009.

Table S14. Out of sample prediction (Manchester to Newcastle) of *g* from regional volumetric MRI data, controlling for total brain volume.

|  |  | **Est.** | **SE** | ***p*** | **ci.lower** | **ci.upper** |
| --- | --- | --- | --- | --- | --- | --- |
| ***Correction A*** |  |  |  |  |  |  |
| Cortical | Train | 0.189 | 0.042 | <0.001 | 0.121 | 0.201 |
|  | Test | 0.189 | 0.042 | <0.001 | 0.106 | 0.272 |
|  |  |  |  |  |  |  |
| Subcortical | Train | 0.252 | 0.020 | <0.001 | 0.212 | 0.292 |
|  | Test | 0.198 | 0.045 | <0.001 | 0.111 | 0.286 |
|  |  |  |  |  |  |  |
| ***Correction B*** |  |  |  |  |  |  |
| Cortical | Train | 0.216 | 0.012 | <0.001 | 0.192 | 0.240 |
|  | Test | 0.157 | 0.026 | <0.001 | 0.106 | 0.208 |
|  |  |  |  |  |  |  |
| Subcortical | Train | 0.270 | 0.013 | <0.001 | 0.245 | 0.296 |
|  | Test | 0.241 | 0.028 | <0.001 | 0.186 | 0.296 |

*Note.* Standardised estimates (Est.), standard errors (SE) and 95% CIs (ci.upper and ci.lower) are reported. Correction A: associations between *g* and composite weighted scores were corrected for total brain volume; Correction B: each ROI is corrected for total brain volume within the training MIMIC model which is used to establish the beta weights.


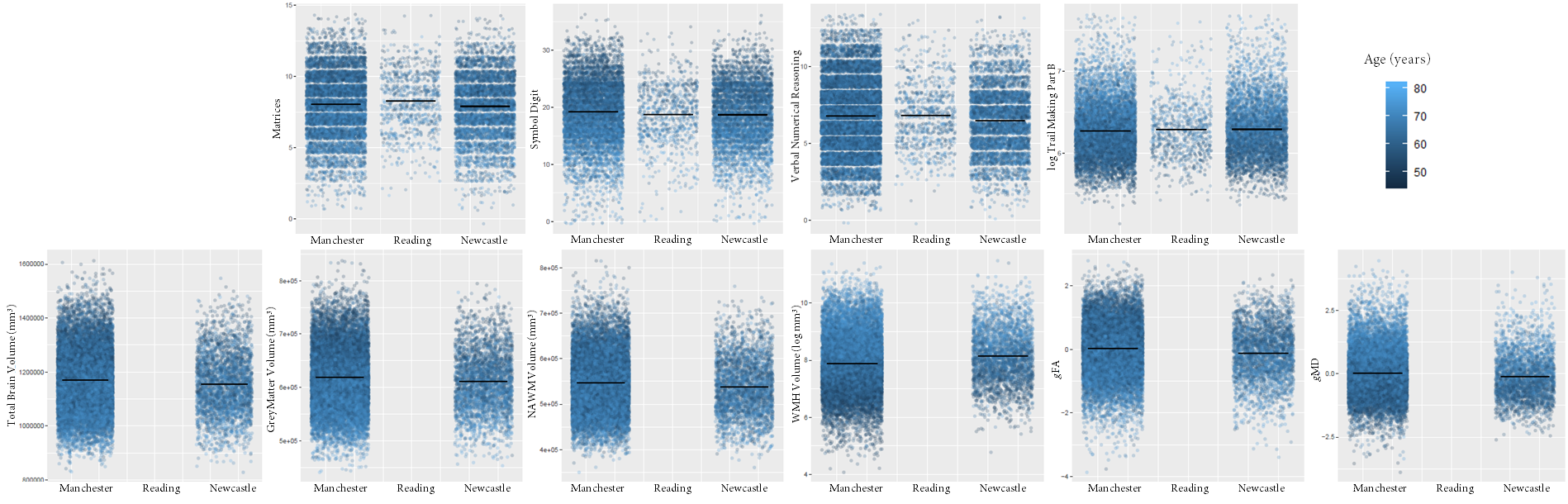
*Figure S1.* Cognitive and MRI measures across UK Biobank assessment centres. Black horizontal lines denote group means; horizontal jitter added to aid visualisation.


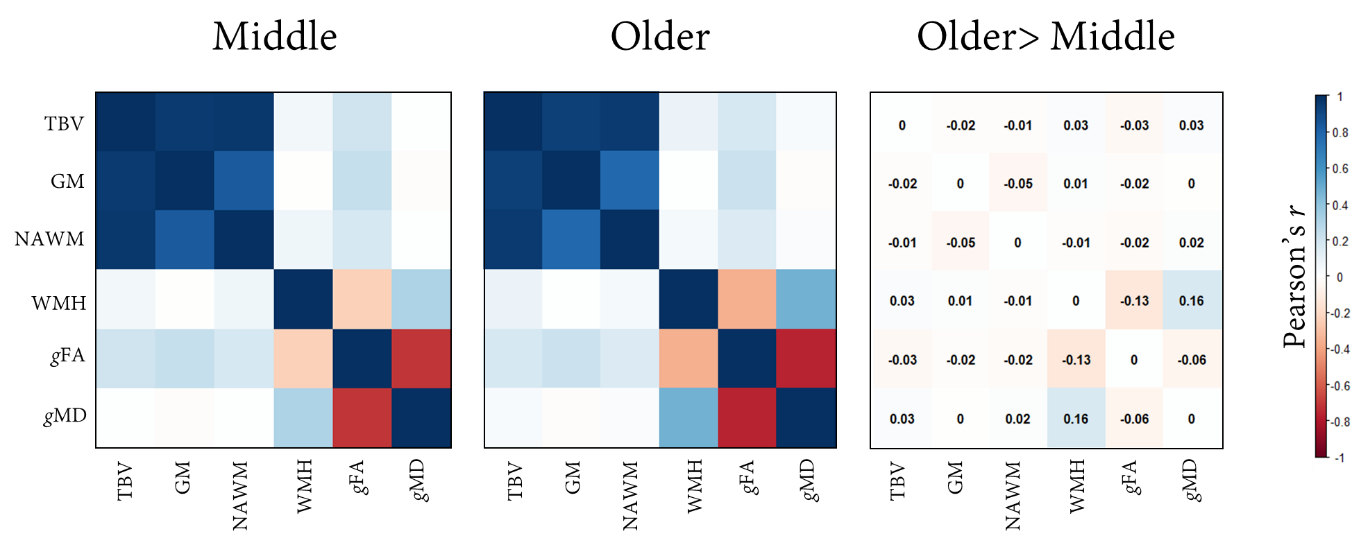


*Figure S2.* Associations among global brain images markers in middle and older age, and the difference in the magnitude of associations. TBV: total brain volume, GM: grey matter volume, NAWM: normal-appearing white matter, WMH: white matter hyperintensity volume, *g*FA: general fractional anisotropy, *g*MD: general mean diffusivity.
